# Comparative genomics: Dominant coral-bacterium *Endozoicomonas acroporae* metabolizes dimethylsulfoniopropionate (DMSP)

**DOI:** 10.1038/s41396-020-0610-x

**Published:** 2020-02-13

**Authors:** Kshitij Tandon, Chih-Ying Lu, Pei-Wen Chiang, Naohisa Wada, Shan-Hua Yang, Ya-Fan Chan, Ping-Yun Chen, Hsiao-Yu Chang, Yu-Jing Chiou, Ming-Shean Chou, Wen-Ming Chen, Sen-Lin Tang

**Affiliations:** 1 Biodiversity Research Center, Academia Sinica, Taipei, 115 Taiwan; 2 Bioinformatics Program, Institute of Information Science, Taiwan International Graduate Program, Academia Sinica, Taipei, 115 Taiwan; 3 Institute of Molecular and Cellular Biology, National Tsing Hua University, Hsinchu, 300 Taiwan; 4 Institute of Fisheries Science, National Taiwan University, Taipei, 10617 Taiwan; 5 Institute of Environmental Engineering, National Sun Yat-sen University, Kaohsiung, 80424 Taiwan; 6 Institute of Oceanography, National Taiwan University, Taipei, 10617 Taiwan; 7 Laboratory of Microbiology, Department of Seafood Science, National Kaohsiung Marine University, No. 142, Hai-Chuan Rd, Nan-Tzu, Kaohsiung City, 811 Taiwan

**Keywords:** Bacterial genomics, Environmental microbiology

## Abstract

Dominant coral-associated *Endozoicomonas* bacteria species are hypothesized to play a role in the coral sulfur cycle by metabolizing dimethylsulfoniopropionate (DMSP) into dimethylsulfide (DMS); however, no sequenced genome to date harbors genes for this process. In this study, we assembled high-quality (>95% complete) draft genomes of strains of the recently added species *Endozoicomonas acroporae* (Acr-14^T^, Acr-1, and Acr-5) isolated from the coral *Acropora sp*. and performed a comparative genomic analysis on the genus *Endozoicomonas*. We identified DMSP CoA-transferase/lyase—a *dddD* gene homolog in all sequenced genomes of *E. acroporae* strains*—*and functionally characterized bacteria capable of metabolizing DMSP into DMS via the DddD cleavage pathway using RT-qPCR and gas chromatography (GC). Furthermore, we demonstrated that *E. acroporae* strains can use DMSP as a carbon source and have genes arranged in an operon-like manner to link DMSP metabolism to the central carbon cycle. This study confirms the role of *Endozoicomonas* in the coral sulfur cycle.

## Introduction

Coral reefs are one of the most diverse ecosystems on Earth, with over 800 different coral species known to date. Of these, corals in the genus *Acropora* are some of the most abundant reef-building corals across the Indo-Pacific region [1]. They are also a significant producer of dimethylsulfoniopropionate (DMSP) [2, 3], an organosulfur compound abundant in animals that harbor symbiotic algae such as scleractinian corals and giant clams [2]. DMSP is present in coral tissues, mucus, and endosymbiotic dinoflagellates (Symbiodiniaceae) [4, 5]. In marine algae, DMSP protects against various stresses, such as oxidative and osmotic stress [6]. Moreover, DMSP also acts as an attractant for specific bacterial groups that have been reported to be part of coral-associated bacterial communities and underpin coral health [7].

Once released from marine planktonic dinoflagellates, most DMSP emanates to surrounding water where it is readily available for microbial catabolic conversion as a source of reduced carbon and sulfur [8, 9]. DMSP is a central molecule in the marine sulfur cycle, and is degraded by bacteria via two pathways: a cleavage pathway and a demethylation pathway [9, 10]. The majority (~75%) of the DMSP is metabolized via the demethylation/demethiolation pathway, producing methylmercaptopropionate [11–13]. The cleavage pathway, which accounts for the remaining ~25% of DMSP, produces dimethylsulfide (DMS)—a climate-active gas and acrylic acid [13–15]. Several DMSP lyases have been identified to date—including DddP, DddY, DddQ, DddW, DddK, and DddL—in organisms like *Rhodobacterales*, *Roseobacters*, *Sulfitobacter*, and *Pseudomonadales* [9, 13, 16]. Another gene, *dddD*, was identified in *Marinomonas* sp., which cleaves DMSP to produce DMS and 3-hydroxypropionate (3-HP)—not acrylate, which is produced by other genes [13]. Moreover, DMSP metabolism yielding DMS and acrylate was also recently identified in coccolithophore algae [17]. The DMS produced after DMSP cleavage is then released into the surrounding water [12].

Coral-associated bacterial communities are highly diverse and dynamic [18–21]. These bacterial communities can be found in various niches associated with corals like coral mucus [22], spaces in the skeleton [23–25], and coral tissues [26–28]. Raina et al. [8] confirmed that coral-associated bacteria have the potential to metabolize organic sulfur compounds present in the coral tissues. They inferred that the majority of the DMSP-degrading bacteria belong to class *Gammaproteobacteria*, including *Alteromonas*-*, Arhodomonas-, Idiomarina-, Pseudomonas-*, and *Spongiobacter (Endozoicomonas)*-related organisms. Of these, *Arhodomonas-, Pseudomonas-*, and *Roseobacter-*related species harbor DMSP-metabolizing genes.

Of all the organisms in the tremendously diverse coral holobionts, genomes of *Endozoicomonas* species have been widely studied for their functional and ecological roles [29, 30]. *Endozoicomonas* species were found to be abundant in the coral holobionts across the globe [31] and are hypothesized to be potential indicators of coral health [32]—they have high abundance in healthy corals and a relatively low abundance in diseased or stressed corals [33–35]. Furthermore, nearly complete and draft genomes of *Endozoicomonas* species isolated from corals and other marine invertebrates [29, 36, 37] have recently been assembled. First, a near-complete genome of the *Endozoicomonas* isolate *Endozoicomonas montiporae* CL-33^T^ from the encrusting pore coral *Montiporae aequituberculata* has provided insights into how this bacterium interacts with its host both outside and inside the coral cell with the help of potential effector proteins [30]. A recent comparative genomics analysis also identified a high number of Type III secretion system (T3SS)-related genes and suggested that (1) most gene ontology terms are associated with the generic transport of molecules and (2) genomes of *Endozoicomonas* species show high plasticity [30]. *Endozoicomonas* species have been hypothesized to play a role in the coral sulfur cycle by effectively metabolizing DMSP into DMS [38, 39]. However, no study has confirmed the genus’ role and no sequenced genome has been found to harbor genes related to this process [30]. Hence, the role of this coral endosymbiont in the coral sulfur cycle remains elusive.

In this study, we assembled high-quality draft genomes of newly added *Endozoicomonas acroporae* strains and profiled their abundances in different coral species in the Indo-pacific region. We identified and functionally characterized a DMSP Co-A transferase/lyase, encoded by a *dddD* homolog, in all sequenced *E. acroporae* genomes. This gene was not present in any other *Endozoicomonas* genomes used in this study. Furthermore, we provide conclusive evidence that *E. acroporae* has a role in the coral sulfur cycle by effectively metabolizing DMSP into DMS and can use DMSP as a carbon source for growth and survival.

## Materials and methods

### Culturing and whole-genome sequencing of *E. acroporae* Acr-1, Acr-5, and Acr-14^T^

Strains of *E. acroporae* Acr-1, Acr-5, and Acr-14^T^ were isolated from the coral *Acropora sp*. from Kenting, off the southern coast of Taiwan, and cultured using a method described previously [40]. Genomic DNA was isolated using the cetyltrimethylammonium bromide method [41]; the quality of the isolated DNA was assessed using NanoDrop 1000 (Thermo Scientific, USA). High-quality DNA was sent to the core sequencing facility at Biodiversity Research Center, Academia Sinica, Taiwan for whole-genome sequencing on the Illumina Miseq platform, with a TRUSeq DNA paired-end library generated to achieve an insert size of 500 bp.

### Genome assembly and annotation

Reads obtained from Illumina MiSeq were quality-filtered and trimmed (Phred score ≥ 30) using NGS QC toolkit v2.3.3 [42]. Quality-filtered and trimmed reads were de novo assembled using CLC Genomics Workbench version 1.10.1 (Qiagen) with a bubble size of 40 and automatic word size enabled. Minimum contig length was set to ≥500 bp (no scaffolding was performed). Assembled genomes were quality checked for completeness, contamination, and heterogeneity using CheckM [43]. Other *Endozoicomonas* species genomes were downloaded from the NCBI genomes database (last accessed January 2018). Gene prediction on all genomes used in this study was performed with Prodigal [44] wrapped in Prokka [45] with default settings to make GFF files compatible for down-stream pan-genome analysis with Roary [46]. Furthermore, genome annotations and up-to-date higher level-functional categories at subsystem levels were obtained from rapid annotation using the subsystem technology (RAST) server [47] with predicted gene calls preserved (February 2018).

### Identification of genomic characteristics

In silico genome–genome distances (GGDs) among the genomes of genus *Endozoicomonas* were calculated using the GGD calculator from the DSMZ server [48], and the average nucleotide identity (ANI) (https://enve-omics.ce.gatech.edu/ani/) calculator was used to calculate the ANI values. Furthermore, amino acid identity (AAI) among the genomes was calculated with CompareM (https://github.com/dparks1134/CompareM). All plots were generated with R (R Core Team, 2016) [49] using the ggplot2 [50] package. Clustered regularly interspaced short palindromic repeat (CRISPR) structures in all genomes were identified using Prokka; prophages and phages within the genomes were identified using the PHAge Search Tool (PHAST) [51], which classified the phages as intact, incomplete, or questionable. T3SS proteins were identified by EffectiveT3 [52] using EffectiveDB with the animal classification module and selective (0.9999) restriction value method enabled. Type IV secretion system (T4SS) proteins were predicted using the web-based T4SEpre (beta) (https://effectors.csb.univie.ac.at/effective) with a minimum score of 0.05. Insertion sequence (IS) elements in the genomes of *E. acroporae* strains and *E. montiporae* were identified using the ISfinder database (https://www.is.biotoul.fr) with blastn and an *e*-value threshold of 1e−5. Furthermore, proteins with eukaryotic repeat domains (Ankyrin repeats (ARPs) and WD40 domain) were identified in all *E. acroporae* strains using the web-based Batch Conserved Domain-Search (CD-search) tool [53] with the CDD-52910 PSSMs database and an *e*-value threshold of 1*e*-5 (October 2019).

### 16S rRNA gene phylogenetic analysis

To determine robust phylogenetic relationships within the genus *Endozoicomonas*, all available 16S rRNA sequences (68 total) were downloaded from the NCBI taxonomy database, for which host information was available to understand the distribution of *Endozoicomonas* species in different marine invertebrates and identify the position of *E. acroporae* strains within the genus *Endozoicomonas*. Sequences were aligned using cmalign from the infernal package [54], which performs a covariance model (CM)-guided SSU rRNA alignment. The CM model for domain bacteria was obtained from the rfam database [55]. A maximum likelihood (ML) phylogeny was computed using IQ-TREE v1.6.11 [56] with the TIM3+F+I+G4 model (Bayesian Information Criterion values, Supplementary Table S1) and 1000 bootstraps. ModelFinder [57] was invoked to automatically select the best model in IQ-TREE [56]. A consensus tree was visualized in iTOL v4 [58].

### *E. acroporae* distribution and abundance in different coral species from the Indo-Pacific region

We analyzed microbial community data publically available from three different studies—1) our laboratory’s previous study [34]; (2) a study of coral-associated bacterial communities in the Red Sea [59]; and (3) coral-associated bacterial community from reefs in the east and west coast of Australia, including Ningaloo Reef, Lizard Island, reefs from the northern sector of the Great Barrier Reef, and Lorde Howe Island [60]—to profile the abundance of *E. acroporae* strains in different coral species from Penghu Archipelago, Taiwan; the Red Sea, Saudi Arabia; and east and west Australia. All three studies used the same operational taxonomic unit (OTU) clustering threshold of 97% and the Greengenes database (v99) [61] for taxonomic assignment. OTU abundance profiles and their representative sequences were downloaded from supplementary materials in Shiu et al. [34], Ziegler et al. [59], and Pollock et al. [60]. We performed similarity searches on all OTU sequences from the three studies against the in-house blast [62] database of all available *Endozoicomonas* 16S rRNA gene sequences with standalone blastn [62] and profiled the relative abundance of *E. acroporae* strains at the three locations with *e*-value < 1e–5 and identity threshold ≥ 99%. A map was drawn using the Generic Mapping Tool [63].

### Comparative genomics: pan-genome analysis and core genome phylogeny

Pan-genome analysis was performed with Roary [46] using GFF files of all the genomes, including *Parendozoicomonas haliclonae* S-B4-1U^T^ (outgroup), obtained from Prokka [45]. Core genome identification and alignment were performed on all genomes using the parameters *-i 80, -e, –n –cd 90*. A Bayesian inference phylogenetic tree was constructed with MrBayes [64] run for one-million generations using the GTR+F+I+G4 (Supplementary Table S1) model, pruning the initial 25% of trees. The model was selected automatically by ModelFinder [57] wrapped in IQ-TREE [56]. An alternate ML tree was also constructed with 1000 bootstraps using IQ-TREE [56]. Trees were visualized with iTOL v4 [58].

### Identification of stress response genes, *dddD CoA-transferase/lyase*, and DMSP metabolism-related operon

The Stress Response Subsystem was analyzed for the distribution of different categories of stress-responsive genes present in the genomes of *Endozoicomonas*. The Sulfur Metabolism Subsystem in the RAST analysis annotated a *dddD* gene capable of metabolizing DMSP into DMS within the “sulfur metabolism—no subcategory”. Furthermore, the presence of domains in the *dddD* gene and DMSP metabolism-related operon genes was determined with a web-based CD-Search [53], NCBI, with default parameters. In addition, we performed a similarity search to annotate DMSP demethylation pathway (sulfur metabolism) genes using the web-based blastkoala [65]. Other DMSP lyases were similarity searched against full-length seed sequences obtained from the pfam database (PF16867) [66] using blastp [62] for all *Endozoicomonas* genomes with *e*-value < 1e–5 and percent identity ≥ 50%. We also performed a phylogenetic analysis on proteins involved in the DMSP metabolism-related operon (see Supplementary Data: Materials and Methods, Supplementary Fig. S8 and Supplementary Table S9).

### DMSP degradation by strain Acr-14^T^

*Endozoicomonas acroporae* Acr-14^T^ was cultivated on Modified Marine Broth Version 4 (MMBV4) medium [29] with several modifications (Supplementary Table S2) at 25 °C for 48 h. Carbon sources, ~3 mM maltose, and 0.5 mM DMSP were added to the enrichment culture of strain Acr-14^T^ and kept at 25 °C for 24 h (OD_600_ of ∼1.0 after incubation). The enrichment culture was centrifuged at 2000 × *g* for 10 min, and the supernatant was discarded. The enrichment culture pellet was washed with 1 ml fresh minimal medium (Supplementary Table S3) twice to remove the MMBV4 medium containing DMSP. Two experimental groups were made for the test: (1) 1 ml of washed bacteria was resuspended in a 40 ml minimal medium containing 0.2% casamino acid and 1 mM DMSP; (2) 1 ml of washed bacteria was resuspended in a 40 ml minimal medium containing 0.2% casamino acid without DMSP. The cultures were sampled at 0, 16, 24, and 48 h for RT-qPCR.

### Gene expression of *dddD* by RT-qPCR

Total RNA was extracted using a TRI-reagent solution (Invitrogen, Carlsbad, CA, USA). The cultured samples (1–2 ml) were centrifuged for 1 min at 12000 × *g* and 4 °C following the manufacturer’s guidelines. The RNA pellet was air-dried and resuspended in nuclease-free water. Residual DNA was removed using a TURBO DNA-free Kit (Invitrogen). The RNA quality was determined using NanoDrop ND-1000 UV-Vis Spectrophotometer (NanoDrop Technologies). RNA integrity was assessed by electrophoresis on a 1% agarose-guanidine thiocyanate gel. Complementary DNA (cDNA) was synthesized from purified RNA using the SuperScript IV First-Strand Synthesis System for RT-qPCR (Invitrogen) following the manufacturer’s guidelines. For cDNA, RT and non-RT samples were screened for residual DNA contamination using the hypervariable V6V8 region of the bacterial 16S rRNA gene (U968F and U1391R).

A primer pair was made for the *dddD* gene based on the genome data of strain Acr-14^T^ for Quantitative PCR using DNASTAR Lasergene [67] and Primer-BLAST tool on BLAST search (NCBI): *dddD*-F (5′-ACCGCATCGCACCACTCAGG-3′) and *dddD*-R (5′-GGCCCCGGTTGTTTCATCAT-3′). It is important to note that the *dddD* gene sequence is 99.24% identical among *E. acroporae* strains. The endogenous control was performed with the *rpoD* gene using *rpoD*-F (5′-AAGGCGGTGGACAAGTTCG-3′) and *rpoD*-R (5′-GATGGTGCGGGCCTGGTCTG-3′). RT-qPCR assays were carried out using the *Applied Biosystems QuantStudio*^*TM*^ *5* Real-Time PCR System. *The standard cycling program* consisted of cycles of UDG activation at 50 °C for 2 min, initial denaturation activation at 95 °C for 2 min and 40 cycles of denaturation at 95 °C for 10 s and annealing at 60 °C for 40 s using PowerUp SYBR Green Master Mix (Thermo Fisher Scientific, USA). A dissociation step was performed to confirm the specificity of the product and avoid the production of primer dimers. For all reactions, 10 ng of template DNA was added to a reaction of 10 μl. The 10 μl reactions contained 5 μl of PowerUp SYBR Green Master Mix (Thermo Fisher Scientific, USA), 3.4 μl sterilized nuclease-free water, 0.3 μl each of the forward and reverse primers (final conc. 0.3 µM), and 1 μl of DNA template. Each sample was performed in duplicate. The relative quantification of the expression ratio was calculated by the comparative 2^−ΔΔCт^ method. Differences between treatments were statistically tested using the *t*-test.

### Quantification of released DMS

To assay the functional activity of the dddD protein in *Endozoicomonas acroporae* strain Acr-14^T^, cultures were first grown in MMB medium with 0.5 mM DMSP for 24 h. After that, 3 ml culture was collected, spun down, and washed twice with minimal medium, and then resuspended with 1 ml minimal medium. This 1 ml culture was then injected into sterile 60 ml vials sealed with a rubber stoppers containing designated medium Treatment (a): 20 ml minimal medium with 0.2% casamino acid and 1 mM DMSP; Treatment (c): 20 ml minimal medium with 0.2% casamino acid, acting as the negative control; and a culture-free Treatment (b): 20 ml minimal medium with 0.2% casamino acid and 1 mM DMSP, acting as the control. Vials were then incubated at 25 °C, 200 rpm, in the dark. After incubation for 0, 24, and 48 h, 1 ml of the headspace air sample was collected and injected into a gas chromatograph (Shimadzu, GC-14B) fitted with a flame ionization detector (150 °C) and column (SGE, 60 m × 0.53 mm ID BP624 × 3.0 µm) to determine the DMS concentration.

### *E. acroporae* grown with DMSP as the carbon source

*E. acroporae* Acr-1, Acr-5, and Acr-14^T^ and *E. montiporae* CL-33^T^ strains were cultivated on MMB medium (along with 0.1% maltose for *E. montiporae* CL-33^T^) at 25 °C for 72 h. The carbon source, 0.1 mM DMSP, was added to the enrichment culture of *E. acroporae* strains and kept at 25 °C for 24 h (OD_600_ of ~0.3 after incubation). 0.1 mM DMSP and 3 mM maltose were added to the enrichment culture of *E. montiporae* and kept at 25 °C for 24 h (OD_600_ of ~0.8 after incubation). The enrichment culture was centrifuged at 2000 × *g* for 10 min, and the supernatant was discarded. The enrichment culture pellet was washed twice by 1 ml fresh minimal medium. After resuspending with minimal medium, the enrichment cultures were added to the treatments and adjusted to OD_600_ of 0.06. All the treatments were kept at 25 °C, 200 rpm, and the OD_600_ of treatment was recorded after incubating for 24, 48, and 72 h.

*E. acroporae* Acr-14^T^, Acr-1, and Acr-5 were cultivated on a minimal medium with 0.2% casamino acid and 0.1, 1, or 3 mM DMSP to test their ability to use DMSP as a carbon source (three replicates each). Treatments with 0.1, 1, or 3 mM DMSP with 0.2% casamino acid were also used to test whether *E. montiporae* CL-33^T^ can use DMSP. In these treatments, *E. montiporae* CL-33^T^ and *E. acroporae* were also grown on 3 mM maltose.

## Results

### 16S rRNA gene phylogeny and *E. acroporae* abundance profiling in different coral species from the Indo-Pacific region

All three strains of *E. acroporae* had only one copy of the 16S rRNA gene, compared with seven copies in the *E. montiporae* CL-33^T^ genome (Supplementary Table S4) [26]. 16S rRNA gene-based phylogeny clustered sequences reflect the host phylogeny in most cases (Supplementary Fig. S1). We identified separate clades for *E. montiporae* and *E. acroporae* (Supplementary Fig. S1). The closest relative of *E. acroporae* strains was a new species within the same clade, *Endozoicomonas coralli*, whose genome has not been sequenced yet. It is worth noting that the *E. acroporae* and *E. coralli* 16S rRNA gene share high (98.69%) sequence identity.

**Fig. 1:**
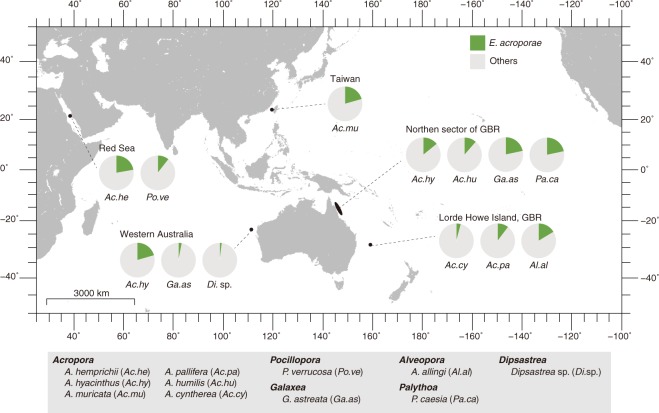
Distribution and abundance profiles of *E. acroporae* strains in the Red Sea, Saudi Arabia; Penghu Archipelago, Taiwan; and eastern and western Australian coral reefs. The green color in pie charts represents relative abundance of *E. acroporae* strains (identified at ≥99% identity and *e*-value < 1e–5) in coral microbial communities as assessed by 16S rRNA amplicon sequencing.

Relative abundance profiles of *E. acroporae* strains were determined (at >99% identity) to obtain an overview of the distribution of this new species in different coral species from three distinct geographical regions (Fig. 1). We determined that *E. acroporae* strains were abundant in corals from the Red Sea, Saudi Arabia (~10–22% of coral bacterial communities); Penghu Archipelago, Taiwan (~20%); and coral reefs in Eastern (~4–22%) and Western (~2–20%) Australia. The presence and relative abundances of *E. acroporae* strains provide evidence that this species is abundant in coral holobionts spread across distinct geographic locations, similar to the distribution of other *Endozoicomonas* species.

### Genome assembly features

Whole-genome sequencing of *E. acroporae* isolates produced genome assemblies with 98.56, 98.99, and 95.25% completeness and contamination levels of 1.39, 2.25, and 2.25% in *E. acroporae* Acr-14^T^, *E. acroporae* Acr-5, and *E. acroporae* Acr-1, respectively (Supplementary Fig. S2). Contigs were checked for similarity against all sequenced Symbiodiniaceae and coral *Acropora* genomes (obtained from www.reefgenomics.org) for contamination, but no significant hit (*e*-value < 1e−5, percent identity ≥ 90, alignment coverage ≥ 50%) was obtained. Thus, our genomes are currently high-quality drafts based on MIMAG standards of completeness and contamination [68]. The *E. acroporae* Acr-1 genome was assembled into 299 contigs with a total of 6.024 Mb, and we predicted 77 tRNAs, 5,144 genes (avg. length: 1002 bp), a gene density of 839 genes per Mb, and 5,059 coding sequences (CDS). *E. acroporae* Acr-5 genome was 6.034 Mb with 295 contigs coding for 80 tRNAs, 5190 genes (avg. length: 904 bp), a gene density of 898 genes per Mb, and 5,101 CDS. The assembled genome of *E. acroporae* Acr-14^T^ was 6.048 Mb with 309 contigs coding for 79 tRNAs, 5104 genes (avg. length: 1014 bp) [37], a gene density of 829 genes per Mb, and 5018 CDS. A list of all the genomes used in this study—along with their sizes (4.049–6.69 Mb) and hosts (coral, sea slug, comb pen shell, and sponge)—is shown in Supplementary Table S5. All *E. acroporae* genomes (Acr-1, Acr-5, and Acr-14^T^) had similar genome sizes (6.024–6.048 Mb) and gas chromatography (GC) contents (49.2, 49.3, and 49.3%, respectively).

### Genomic characteristics of genus *Endozoicomonas*

**Fig. 2:**
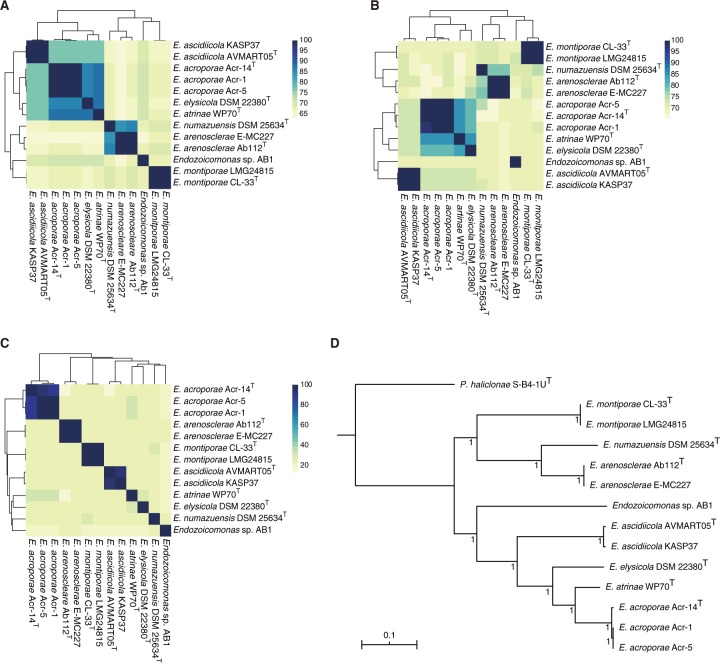
Genomics characteristics of genus *Endozoicomonas*. Heatmaps based on **(a)** average amino acid identity (AAI), **(b)** average nucleotide identity (ANI), and **(c)** genome–genome distance (GGD) for all the sequenced genomes from genus *Endozoicomonas*. **(d)** Core genome (313)-based Bayesian inference phylogenetic tree generated from MrBayes run for one-million generations using the GTR+F+G4 model; posterior probabilities are mentioned at the branch-points. *Parendozoicomonas haliclonae* S-B4-1U^T^ was used as the outgroup.

*Endozoicomonas* species have large genomes ranging from 4.049 Mb (*Endozoicomonas* sp. AB1) to 6.69 Mb (*E. elysicola* DSM22380). The average genome has ~5100 protein-coding genes and a GC content of 47.6%. The average GGD, ANI, and AAI values among the *Endozoicomonas* species were ~46, ~73, and ~75%, respectively, which are at the lower end of the 62–100% range of interspecies variation within a genus [69], indicating high genomic diversity (Fig. 2a–c).

A diverse array of IS elements were identified in genomes of *E. acropora*e and *E. montiporae*. For example, of the total 44 IS elements identified, 22 were only present in *E. acroporae* genomes, while 18 were only present in *E. montiporae* genomes. Relatively few IS elements (ISEc46, ISEcret1, ISEc42, and ISPa18) were present in both species. Each genome also harbored some unique IS elements (Supplementary Fig. S3).

Three *E. acroporae* genomes (Acr-14^T^, Acr-5, and Acr-1) encoded more T3SS effector proteins (523, 499, and 499, respectively) —than other *Endozoicomonas* species (Supplementary Table S6). Moreover, all *Endozoicomonas* species also harbored a large number of T4SS effector proteins within the range of 106 (*Endozoicomonas* sp. AB1) to 226 (*E. ascidiicola* KASP37) (Supplementary Table S6). Interestingly *E. acroporae* genomes also had a high count of proteins containing ARPs and WD40 domains, though most of these were annotated as hypothetical proteins (Supplementary Table S7). We further identified different phage insertions in all *Endozoicomonas* genomes (Supplementary Table S8) and different CRISPR counts in the genomes of *E. acroporae* Acr-14^T^, Acr-5, and Acr-1 (Supplementary Table S4).

### Core genome phylogeny

Core gene (*n* = 313)-based phylogenetic analysis reflected the host phylogeny in some cases, and also hinted towards a high genomic divergence within the species of genus *Endozoicomonas. Endozoicomonas* genomes isolated from the same host clustered very tightly together, e.g. *E. acroporae* and *E. montiporae* with the coral host, and *E. ascidiicola* and *E. arenosclerae* with a sponge host (Fig. 2d and Supplementary Fig. S4). Moreover, *E. numazuensis* and *E. arenosclerae* genomes shared a branch, and were both isolated from the sponge. *E. acroporae* Acr-1 and Acr-5 clustered tightly while sharing a branch with *E. acroporae* Acr-14^T^, which confirms that Acr-1 and Acr-5 are closer to each other (ANI: 100%) than to Acr-14^T^ (ANI: 98.10% and 99.02%, respectively).

### Analyzing RAST subsystems

RAST subsystems—carbohydrates, protein metabolism, and amino acids and derivatives—had the highest number of genes, representing an average of 11.69%, 10.85%, and 13.29% of all the annotated genes, respectively (Supplementary Fig. S5), in all *Endozoicomonas* genomes. We focused our analysis on two other important subsystems: stress response and sulfur metabolism. The highest number of genes were annotated for oxidative stress (39.16% of stress genes) in the stress response subsystem, followed by unclassified (16%) and detoxification stresses (12.53%) (Supplementary Fig. S6). Interestingly, we identified a *dddD* gene homolog involved in sulfur metabolism in the *E. acroporae* Acr-14^T^, Acr-5, and Acr-1 genomes. This gene was not present in other *Endozoicomonas* genomes (Supplementary Table S5). Furthermore, no other genes involved in DMSP metabolic pathways were identified in any *Endozoicomonas* genomes. The *dddD* gene present in *E. acroporae* genomes has two identical CaiB domains (positions: 1–416, 439–821) belonging to the coenzyme-A transferase superfamily (Supplementary Fig. S7).

### DMSP metabolism operon and links to the central carbon cycle

**Fig. 3:**
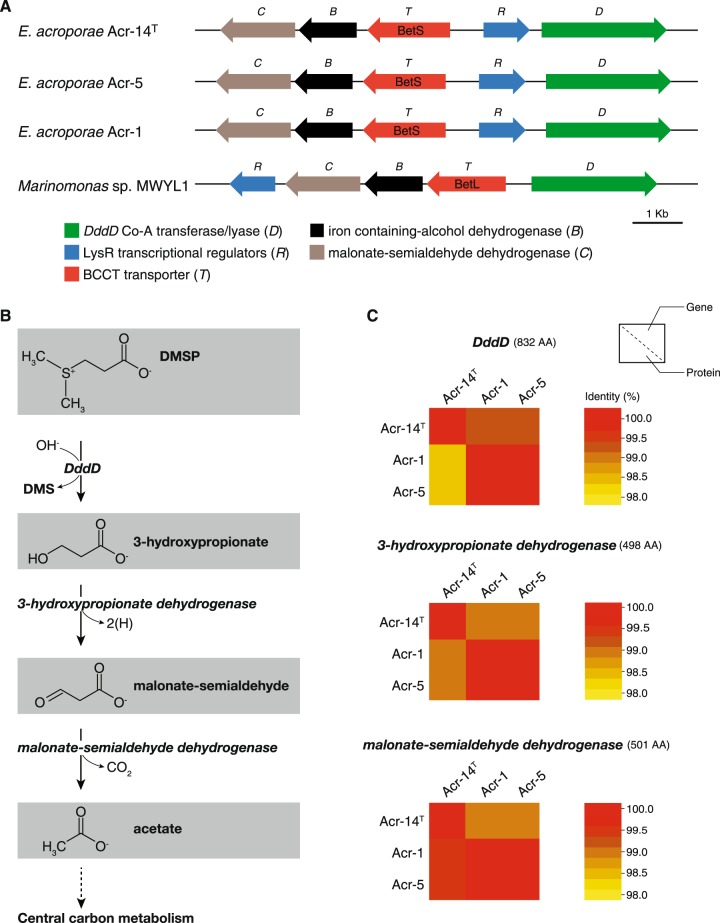
Linking DMSP metabolism to the central carbon cycle in *E. acroporae*. **(a)** DMSP metabolism operon, with genes arranged based on the layout of the operon within all *E. acroporae* strains, with a similar arrangement in *Marinomonas* sp. MWYL1 and different putative DMSP transporters (*BetS* and *BetL*). **(b)** The metabolic pathway represented by the genes, linking DMSP metabolism to the central carbon cycle, producing acetate from DMSP when DMS is released in *E. acroporae*. **(c)** Heatmaps based on gene/protein identity for the three enzymes (from all *E. acroporae* strains) used in the metabolic cycle: DddD, 3-hydroxypropionate dehydrogenase (EC 1.1.1.59), and malonate-semialdehyde dehydrogenase (EC 1.2.1.18).

We identified homologs of the DMSP transcriptional regulator (LysR family), a sulfur transporter belonging to the betaine/carnitine/choline transporter family hypothesized to transport DMSP and genes involved in producing acetate from DMSP (Fig. 3a). We discerned a complete pathway with genes arranged in a consecutive manner to form an operon that can yield acetate from DMSP metabolism via three-step enzymatic reactions mediated by DddD, 3-HP dehydrogenase (EC 1.1.1.59) and malonate-semialdehyde dehydrogenase (EC 1.2.1.18) (Fig. 3b) in all the *E. acroporae* genomes, with significant gene and protein identity for all the three genes (>97%) and proteins (>97%) (Fig. 3c). Identification of the DMSP cleavage pathway leading to central carbon metabolism suggested that *E. acroporae* species can use DMSP as a carbon source. Furthermore, DMSP metabolism-related proteins of *E. acroporae* formed distinct clades in phylogenetic analysis (Supplementary Fig. S8) suggesting presence of high diversity in these genes across different bacteria.

### *dddD* gene activity and DMS quantification

**Fig. 4:**
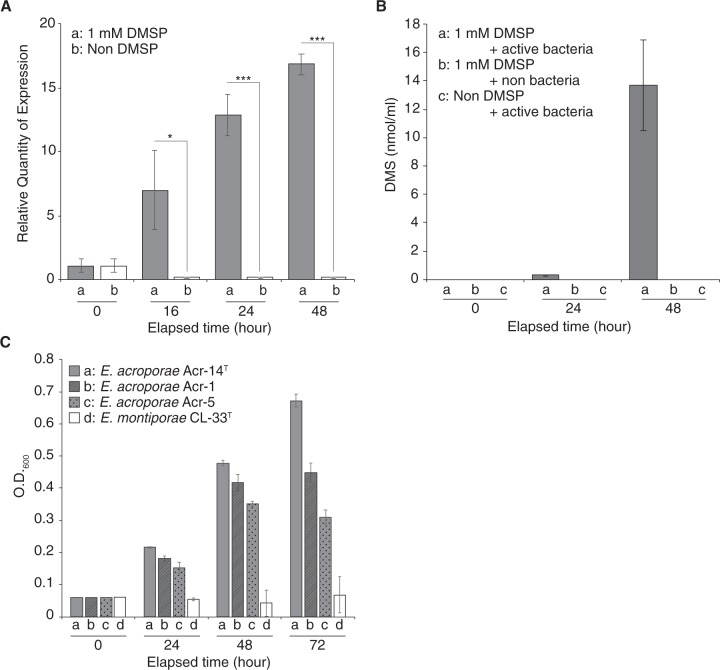
Functional analysis of *dddD* gene, DMS quantification, and ability of *E. acroporae* to use DMSP as carbon source. **(a)** RT-qPCR of the *dddD* gene from *E. acroporae* Acr-14^T^ to measure temporal expression change in the presence of 1 mM DMSP (Student’s *t* test: *p < 0.05, ***p < 0.01). **(b)** GC-based quantification of DMS released from *E. acroporae* Acr-14^T^ cultures in the presence of DMSP (1 mM). **(c)** Growth on 0.1 mM DMSP; *E. acroporae* strains can use DMSP as its carbon source, whereas *E. montiporae* CL-33^T^ did not show signs of using DMSP as its carbon source.

RT-qPCR was used to examine the expression of the *dddD* gene in *E. acroporae* Acr-14^T^. *dddD* gene expression increased with sampling time in the condition with 1 mM DMSP (Fig. 4a). *dddD* gene expression in this condition was 42.77, 56.52, and 91.37 times higher than samples without DMSP at 16, 24, and 48 h, respectively (*t*-test*, p* < 0.05), confirming that *dddD* was active in *E. acroporae*. After confirming the *dddD* gene expression, we quantified the amount of DMS released by *E. acroporae* when incubated in a DMSP-rich environment with a time series detection (0, 24, and 48 h). The DMS signal could only be detected in Treatment (a), and there was no DMS signal in the control groups (Treatments (b) and (c)) (Fig. 4b). A temporal increase in released DMS concentration in Treatment (a) confirmed the ability of *E. acroporae* to metabolize DMSP into DMS.

### DMSP as the carbon source

*E. acroporae* strains grew on 0.1 and 1 mM DMSP, but showed no signs of growth on 3 mM DMSP (Fig. 4c and Supplementary Fig. S9), suggesting that it is able to use only a certain range of DMSP as its carbon source. In 0.1 mM DMSP, Acr-14^T^, Acr-1, and Acr-5 had a mean OD_600_ value of ~0.67, ~0.45, and ~0.31, respectively, after 72-h of incubation (Fig. 4c). In contrast, *E. montiporae* CL-33^T^ cells appeared to form aggregates in all DMSP concentrations after 24 h (Supplementary Fig. S10) and were visualized with viability staining (details in Supplementary Data: Material and Methods). These result showed that *E. montiporae* CL-33^T^ formed aggregates, making OD_600_ measurements unreliable and suggesting that *E. montiporae* CL-33^T^ can use maltose, but not DMSP, as its carbon source (Supplementary Fig. S10). *E. acroporae* Acr-14^T^ also used maltose for growth (Supplementary Fig. S10).

## Discussion

In this study, we performed genomic (and comparative genomic) analyses, functional assays, and abundance profiling of strains from a new species *E. acroporae* in the marine bacterial genus *Endozoicomonas*. We assembled high-quality draft genomes of *E. acroporae* strains and identified the *dddD* gene involved in DMSP metabolism in all *E. acroporae* strains. Furthermore, we characterized the functional activity of the gene and the ability of *E. acroporae* strains to grow on DMSP as a carbon source. This is the first study to establish genomic and functional evidence that *Endozoicomonas* species play a role in the coral sulfur cycle.

### DMSP breakdown by *E. acroporae* and its utilization for growth and survival

*Endozoicomonas* species have been suggested to play a role in the coral sulfur cycle via DMSP metabolism [8, 70]. However, no gene related to DMSP metabolism was found in previously sequenced *Endozoicomonas* genomes (Supplementary Table S5) [30]. Furthermore, using enrichment cultures from coral mucus, tissue, and skeleton, certain bacterial species have been reported to degrade DMSP, including *Spongiobacter (Endozoicomonas) nickelotolerans*, but no gene related to this function was identified, and it was hypothesized to use a different pathway for DMSP degradation [8]. In this study, we investigated the sulfur metabolism pathway in high-quality draft genomes (Supplementary Fig. S2) of *E. acroporae* strains and identified a *dddD* gene that codes for a bi-functional enzyme with Co-A transferase/lyase activity that cleaves DMSP into volatile DMS [9, 71]. Among the eight identified DMSP lyases in different bacterial species, only *dddD* produces 3-HP and DMS, whereas others form acrylate and DMS [72]. 3-HP is not the usual product of other DMSP lyases, which directly produce acrylate after the cleavage due to their unusual organization of class III Co-A transferase domains [73]. Co-A transferase domains present in the DddD protein are similar to CaiB (Supplementary Fig. S7), which is a homodimer capable of adding a Co-A to carnitine [13], a biochemical analysis showed that DddD is unlikely to be a carnitine CoA-transferase [71]. RT-qPCR-based temporal analysis of *E. acroporae* strains in a DMSP-rich environment showed that *dddD* activity increased significantly (Fig. 4a); moreover, the GC analysis also showed that high amounts of DMS were released, which is evidence that *dddD* is functionally active (Fig. 4b).

Genomic analysis also identified genes arranged in consecutive order to form an operon with DMSP transport, metabolism, and transcriptional regulator genes (Fig. 3a) in all the strains of *E. acroporae*. *dddT* is a transporter that imports molecules like DMSP [13, 74], and thus has been suggested to import DMSP. *dddR* is a transcription regulator able to activate the expression of the *dddD* gene in response to DMSP [13]. The functions of *dddB* and *dddC* have not been characterized biochemically, but they are hypothesized to be involved in oxido-reductive functions based on their sequence similarities to other oxidoreductases, which modify DMSP either before or after the addition of the acyl CoA moiety by *dddD* [13]. Interestingly, the arrangement of genes around *dddD* is of a ‘pick ‘n’ mix’ form where, in some cases, corresponding genes have very similar sequence identity and the same function, but in others, overall corresponding gene sequences are very different but may have the same function and mechanism [74]. This arrangement is present in several DddD^+^ bacteria, like *Marinomonas sp*. MWYL1, *Sagittus* sp. E37, and *B. cepacia* AMMD [74]; although a similar gene arrangement was observed between *Marinomonas sp*. MWYL1 and *E. acroporae* strains (Fig. 3a).

The ability of *E. acroporae* strains to use DMSP as their carbon source (Fig. 4c, Supplementary Fig. S9)—unlike *E. montiporae*, which is unable to utilize DMSP, even at higher concentrations (Supplementary Fig. S10)—is further evidence that *E. acroporae* strains can metabolize DMSP and use it for growth and survival. This finding indicates that DMSP metabolism is linked to the central carbon cycle in these species, as we predicted in the genomic analysis (Fig. 3b). Furthermore, it also confirmed the hypothesis that marine bacteria that harbor *dddD* have the ability to use DMSP as the carbon source [75].

### Genome architecture of *Endozoicomonas* species and putative relationship with the host

Bacterial genome size can reflect its evolutionary dependency on the host when they are engaged in obligate symbiosis [76]. A striking feature of obligate symbionts is that they have smaller genome sizes than facultative symbionts, and lower gene densities than the bacterial average of 85–90% [75, 76]. However, genomes of *Endozoicomonas* species are relatively large, ranging from 4.09 to 6.69 Mb; this includes *E. acroporae* strains, which have an average genome length of ~6.03 Mb, suggesting that they might have a free-living stage. The possibility that *Endozoicomonas* species might have a free-living stage is further supported by their large number of coding genes (avg. ~5000 proteins) and average gene density of ~855 genes per Mb (Supplementary Table S4) [77, 78]. These features suggest that genome streamlining [79], a notable feature of symbiotic bacteria, is not prominent in genus *Endozoicomonas*. Moreover, a diverse array of phages (Supplementary Table S8) and IS elements (Supplementary Fig. S3) provides clues about the infection and colonization histories of different marine hosts, aided by frequent divergence events.

A recent study by Ding et al. [29] reported a high proportion of repeat sequences and a variety of IS elements in the genome of *E. montiporae*, which may help the bacterium adapt to the host and also identified the *N-deglycosylation* enzyme that helps penetrate the mucus layer of the host. These findings suggest that *Endozoicomonas* can transition between different symbiotic lifestyles. We identified a high number of eukaryotic repeat proteins in *E. acroporae* genomes (Supplementary Table S7); our results are similar to a recent study on *Porites lutea* microbial symbionts, which identified >50 copies of eukaryotic repeat proteins in a metagenome-derived *Endozoicomonas* genome [80], suggesting a symbiotic relationship. Furthermore, we also identified a high count of secretory (T3SS and T4SS) proteins in the genomes of *E. acroporae* strains (Supplementary Table S6) that may help transport organic macromolecules and effector proteins between the host and symbiont. Both T3SS and T4SS proteins help bacteria interact with their host [81, 82], and our study identified a complete gene set for the assembly of these vital secretory systems. *E. acroporae* has secretory genes that regulate host mechanisms, similar to those of *E. montiporae*, and can increase the chances that bacteria survive in the host while improving the host fitness [29]. In addition, other genes found in *E. acroporae* may also provide clues about the survival strategy of *E. acroporae* in hosts. For example, identification of a catalase gene in *E. acroporae* strains—along with phosphoenolpyruvate synthase and 7,8-dihydro-8-oxoguanine triphosphatase as secretory genes from T3SS—can help the bacterium survive by scavenging H_2_O_2_, modulating the gluconeogenesis, and confer resistance from oxidative stresses to the host [29, 83, 84]. Upregulating genes involved in gluconeogenesis have been proposed as a response to stress-induced starvation in corals [84].

Phylogenetic analysis based on a core genome (*n* = 313) clustered the species to reflect their host phylogeny (Fig. 2d), similar to results obtained in earlier studies [30, 31, 60]. However, even when the host type was the same (i.e. stony coral), *E. montiporae* and *E. acroporae* did not share any branch, and their strains clustered tightly within their clade, which suggests that the co-diversification between host and symbiont is complex in nature (Fig. 2d and Supplementary Fig. S4). Moreover, a highly reduced core genome and similar ANI, GGD, and AAI at the lower end of interspecies range give clues about the diversity in genus *Endozoicomonas* (Fig. 2a–c).

### Oxidative stress response*, E. acroporae* distribution, and potential implications for DMSP cycling in corals

Reef-building corals, along with soft corals, are prolific producers of DMSP in both temperate and tropical reefs, making them important in DMSP cycling [4, 85]. Furthermore, there is evidence that DMSP helps coral mitigate intracellular oxidative stress and the process considered to initiate bleaching induced mortality [86, 87]. *Endozoicomonas* species have been found to dominate the microbiomes of diverse marine hosts residing in shallow depths or intertidal zones, such as corals in tropical and temperate reefs [88, 89]. These hosts experience changes in environmental conditions such as thermal stress, exposure to ultraviolet radiation, and tidal heights, which lead to oxidative stress and has been shown to influence DMSP upregulation [86, 90]. Interestingly, genomes of *Endozoicomonas* species, including *E. acroporae*, harbor a high percentage of oxidative stress-responsive genes (Supplementary Fig. S6) which provide clues for their potential to mitigate oxidative stress. Furthermore, the conclusion that *E. acroporae*, which is widely distributed in diverse coral genera in the Indo-pacific region (Fig. 1), can metabolize DMSP to DMS suggests that it might play a role in the coral sulfur cycle. Interestingly, this bacterium may also protect its host from the coral pathogen *Vibrio coralliilyticus*, which uses DMSP as a cue to find physiologically stressed corals, but shows no chemoattraction towards DMS [91]. The presence of *E. acroporae* in different coral species also supports the idea that this new species has a broad host range similar to other members of *Endozoicomonas*. Moreover, it also highlights the importance of studying *Endozoicomonas* in greater detail. With only very few cultured isolates known to date, identification of new culturable isolates will enhance our understanding of this diverse marine genus and help establish its functional and ecological roles in a diversity of hosts.

## Conclusion

*Endozoicomonas* species have been long hypothesized to play a role in the coral sulfur cycle, and this study provides the first genomic and functional evidence to support this hypothesis. *E. acroporae* strains cannot only metabolize DMSP to produce DMS, but also use DMSP as a carbon source for growth and survival. In our study, we also identified the first DMSP-related operon in *E. acroporae*, which links DMSP metabolism to the central carbon cycle. Furthermore, the presence of stress-responsive genes at higher proportions gives clues about how this genus adapted in marine environments. Since very few species in this diverse marine genus have been cultured to date, the possibility that there are other genes or mechanisms of DMSP metabolism, along with other functional roles in coral reefs, cannot be ignored. More focus on this genus in regards to coral reef health can provide better insights in the near future.

## Supplementary information

Supplementary Data

Supplementary Figure S1

Supplementary Figure S2

Supplementary Figure S3

Supplementary Figure S4

Supplementary Figure S5

Supplementary Figure S6

Supplementary Figure S7

Supplementary Figure S8

Supplementary Figure S9

Supplementary Figure S10

Supplementary Table S1

Supplementary Table S2

Supplementary Table S3

Supplementary Table S4

Supplementary Table S5

Supplementary Table S6

Supplementary Table S7

Supplementary Table S8

Supplementary Table S9

## Data Availability

Draft genomes of *E. acroporae* Acr-1 and *E. acroporae* Acr-5 have been deposited into GenBank under accession IDs SAUT00000000 and SAUU00000000, respectively. The *E. acroporae* Acr-14^T^ genome was previously made public under accession ID PJPV00000000.
