# Supplementary material for: Comparative genomics: Dominant coral-bacterium Endozoicomonas acroporae metabolizes dimethylsulfoniopropionate (DMSP)
Source: ISME J. 2020 Feb 13;14(5):1290–303. doi: 10.1038/s41396-020-0610-x (PMC7174347; doi:10.1038/s41396-020-0610-x)
Supplement: Supplementary file 1 — Supplementary Data [file 41396_2020_610_MOESM1_ESM.docx]

**Comparative genomics: Dominant coral-bacterium *Endozoicomonas acroporae* metabolizes dimethylsulfoniopropionate (DMSP)**

**Kshitij Tandon^1,2,3^, Chih-Ying Lu^1*^, Pei-Wen Chiang^1*^, Naohisa Wada^1^, Shan-Hua Yang^4^, Ya-Fan Chan^1^, Ping-Yun Chen^5^, Hsiao-Yu Chang^5^, Yu-Jing Chiou^1,6^, Ming-Shean Chou^5^, Wen-Ming Chen^7^, Sen-Lin Tang^1,2#^**

^1^Biodiversity Research Center, Academia Sinica, Taipei 115, Taiwan

^2^Bioinformatics Program, Institute of Information Science, Taiwan International Graduate Program, Academia Sinica, Taipei 115, Taiwan

^3^Institute of Molecular and Cellular Biology, National Tsing Hua University, Hsinchu 300, Taiwan

^4^Institute of Fisheries Science, National Taiwan University, Taipei 10617, Taiwan.

^5^Institute of Environmental Engineering, National Sun Yat-sen University, Kaohsiung 80424, Taiwan, ROC

^6^Institute of Oceanography, National Taiwan University, Taipei 106, Taiwan

^7^Laboratory of Microbiology, Department of Seafood Science, National Kaohsiung Marine University, No. 142, Hai-Chuan Rd, Nan-Tzu, Kaohsiung City 811, Taiwan, ROC.

* These authors contributed equally

# Corresponding author

Corresponding author Email: [sltang@gate.sinica.edu.tw](mailto:sltang@gate.sinica.edu.tw)

**Materials and methods**

**DddD, LysR, and BCCT proteins phylogeny.**

We performed a similarity search for DddD, LysR (dddR), and BCCT (BetS) protein sequences of *E. acroporae* using the web-based UniProt-BLAST (https://www.uniprot.org/blast/) against the “UniProtKB reference proteomes plus Swiss-Prot” database. Sequences from the top 25 hits with e-value <1e-5 and percentage identity >50% were downloaded and aligned in MUSCLE [1] with default parameters. Best-fit evolutionary model selection was performed with ModelFinder (Supplementary Table S9); models were selected with lower Bayesian Information Criterion. A Maximum-Likelihood tree with 1000 bootstrap support values was generated with IQ-TREE. Consensus bootstrap trees were visualized in iTOL v4 (Supplementary Figure S8).

**Viability stain**

A viability stain was performed to visualize aggregates formed by *E. montiporae* CL-33^T^ cells in all DMSP concentrations tested in this study. *E. montiporae* cultures were stained with LIVE/DEAD *Bac*Light bacterial viability lit (L7012, Thermo Fisher Scientific) according to the manufacture's protocol. The stained cells were observed through two band pass filters (FITC [EX 465-495, DM 505, and BA 515-555] and Tx Red [Ex 540-580, DM 595, and BA 600-660]) under a Nikon ECLIPSE 90i (Nikon). Fluorescence microscopy images were taken by both functions of Z-series acquisition and EDF focused document (Z-Map method: Balanced) in NIS-elements AR software (Ver.5.11.02; Nikon).

**Reference**

1. Edgar RC. MUSCLE: multiple sequence alignment with high accuracy and high throughput. Nucleic Acids Res. 2004: 32; 1792-1797.

**Supplementary Figures and Tables Legends**

Supplementary Figure S1. 16S rRNA gene-based phylogenetic tree for all 16S rRNA sequences (68 in number) from genus *Endozoicomonas* whose source of isolation is known. *E. acroporae* strains belong to the clade coral group 1 *Acropora* sp., along with a newly discovered species *Endozoicomonas coralli* strain Acr-12. A maximum-likelihood tree was constructed with the TIM3+F+I+G4 evolutionary model and 1000 bootstraps. Bootstrap support values (rounded to the first decimal place) are mentioned at branch-points.

Supplementary Figure S2. CheckM plots for *Endozoicomonas acroporae* strains Acr-1, Acr-5, and Acr-14^T^.

Supplementary Figure S3. Distribution of different IS elements in *E. acroporae* and *E. montiporae* strains. Color codes represent the different combinations and numbers represent the copies of each IS element identified in the genome.

Supplementary Figure S4. Core-genome (n=313)-based Maximum-Likelihood tree constructed with 1000 bootstraps using the GTR+F+I+G4 model. *Parendozoicomonas haliclonae* S-B4-1U^T^ was used as the outgroup. Bootstrap support values are mentioned at branch-points.

Supplementary Figure S5. The proportion of different subsystem annotations by RAST Server on all the genomes used in this study.

Supplementary Figure S6. The proportion of different stress-responsive genes annotated by RAST in the Stress Response Subsystem. Oxidative stress response genes account for ~40% of the total genes annotated.

Supplementary Figure S7. Representation of functional domains specific hit (CaiB) and superfamily (CoA_transf_3) present in the DddD protein of *E. acroporae* strains.

Supplementary Figure S8. Maximum-Likelihood Trees for DMSP metabolism-related proteins. **A)** Phylogenetic tree for DddD proteins constructed with 1000 bootstraps using the LG+I+G4 model; SWISSPROT ids for proteins are mentioned in parentheses. **B)** Phylogenetic tree for BCCT transporter proteins constructed with 1000 bootstraps using the LG+I+G4 model; SWISSPROT ids for proteins are mentioned in parentheses. **C)** Phylogenetic tree for LysR regulator proteins constructed with 1000 bootstraps using the LG+G4 model; SWISSPROT ids for proteins are mentioned in parentheses. All bootstrap support values (>50, rounded to the first decimal place) are mentioned on branch-points.

Supplementary Figure S9. OD_600_ values as an indicator of *E. acroporae* Acr-1, Acr-5, and Acr-14^T^ growth in **A)** 0.1 mM, **B)** 1 mM, and **C)** 3mM DMSP.

Supplementary Figure S10. OD_600_ values and viability stain results for *E. montiporae* CL-33^T^ grown in 0.1, 1, and 3 mM DMSP and 3 mM maltose. *E. montiporae* CL-33^T^ cells formed aggregates (*agg*.) at all concentrations of DMSP, as shown in both the culture and Live/Dead images*. E acroporae* Acr-14^T^ grew in 0.1 mM DMSP. We observed variation in OD_600_ values of *E. acroporae* Acr-14^T^ in this experiment compared to the carbon source experiment at different concentrations, which is probably because the cells were washed gently by swirling than pipetting after centrifugation and different culture stock and others. Both *E. acroporae* Acr-14^T^ and *E. montiporae* CL-33^T^ grew on 3 mM maltose. Viability staining was used to visualize the cells in all treatments for both cultures with green fluorescence (live cells) and red fluorescence (dead cells).

Supplementary Table S1. Model selection statistics for 16S rRNA and Core-genome based phylogenetic analysis. The best model was selected based on BIC values.

Supplementary Table S2. Composition of Modified Marine Broth, Version 4 (MMBV4*)*.*

Supplementary Table S3. Composition of minimal medium for *Endozoicomonas*.

Supplementary Table S4. Genome assembly characteristics of *E. acroporae* strains (this study).

Supplementary Table S5. List of genomes used in this study for comparative genomic analysis with their genome size, host, and presence of *dddD* gene.

Supplementary Table S6. Count of Type III secretion system (T3SS) and Type IV secretion system genes identified in genomes of *Endozoicomonas* species.

Supplementary Table S7. Ankyrin repeat and WD40 domain-containing proteins identified in the genomes of *Endozoicomonas acroporae* strains.

Supplementary Table S8. Phage insertions identified in genomes of genus *Endozoicomonas*. Only intact phages are annotated.

Supplementary Table S9. Model selection statistics for DddD, LysR, and BCCT protein sequences for phylogenetic analysis. The best model was selected based on BIC values.
