# Supplementary figures and images for: Comparative genomics: Dominant coral-bacterium Endozoicomonas acroporae metabolizes dimethylsulfoniopropionate (DMSP)
Source: ISME J. 2020 Feb 13;14(5):1290–303. doi: 10.1038/s41396-020-0610-x (PMC7174347; doi:10.1038/s41396-020-0610-x)

*E. acroporae* Acr-1

*E. acroporae* Acr-14

*E. acroporae* Acr-5

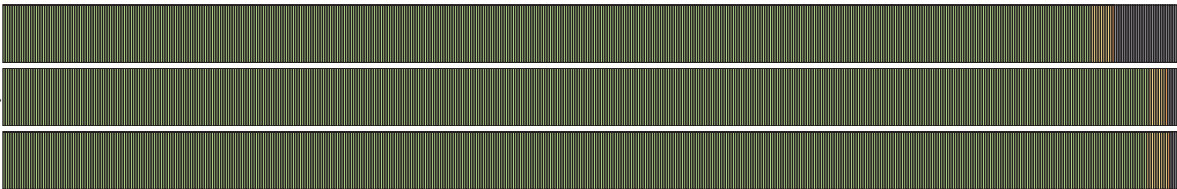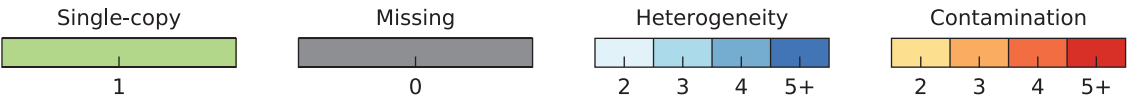

Supplement: Supplementary file 3 — Supplementary Figure S2 [file 41396_2020_610_MOESM3_ESM.pdf]

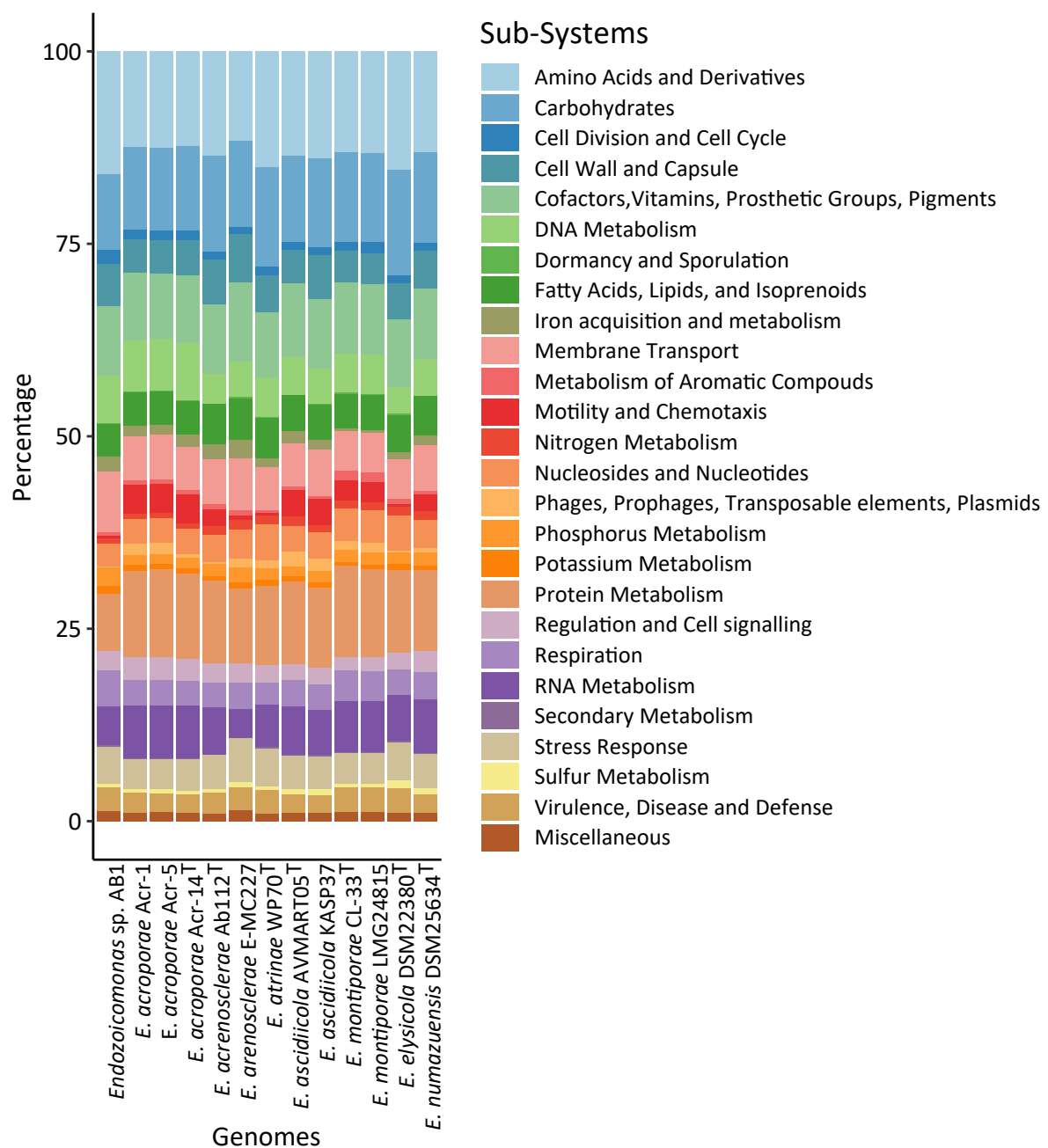

Supplement: Supplementary file 6 — Supplementary Figure S5 [file 41396_2020_610_MOESM6_ESM.pdf]

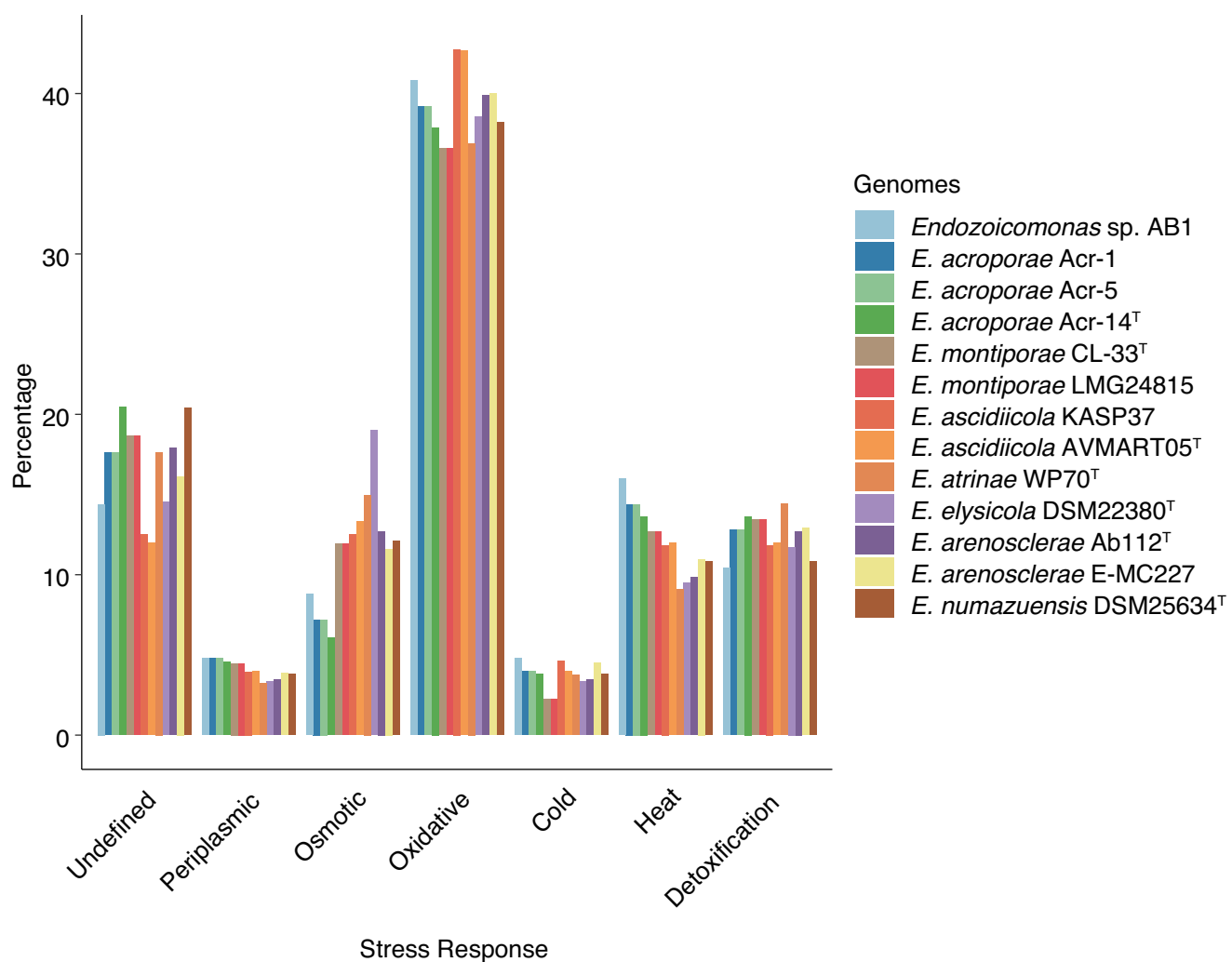

Supplement: Supplementary file 7 — Supplementary Figure S6 [file 41396_2020_610_MOESM7_ESM.pdf]

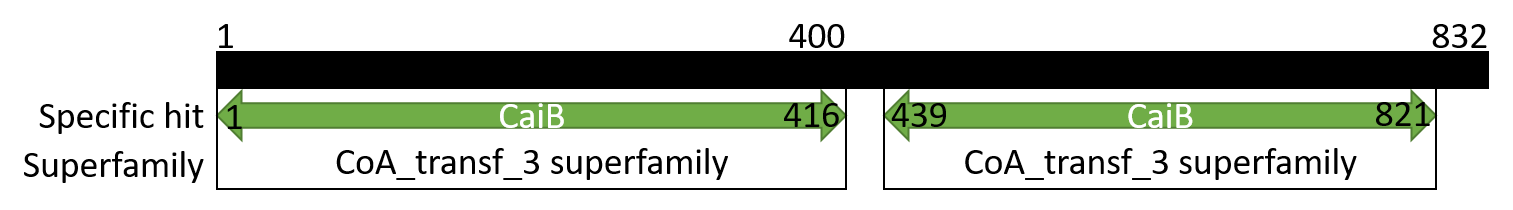

Supplement: Supplementary file 8 — Supplementary Figure S7 [file 41396_2020_610_MOESM8_ESM.tif]

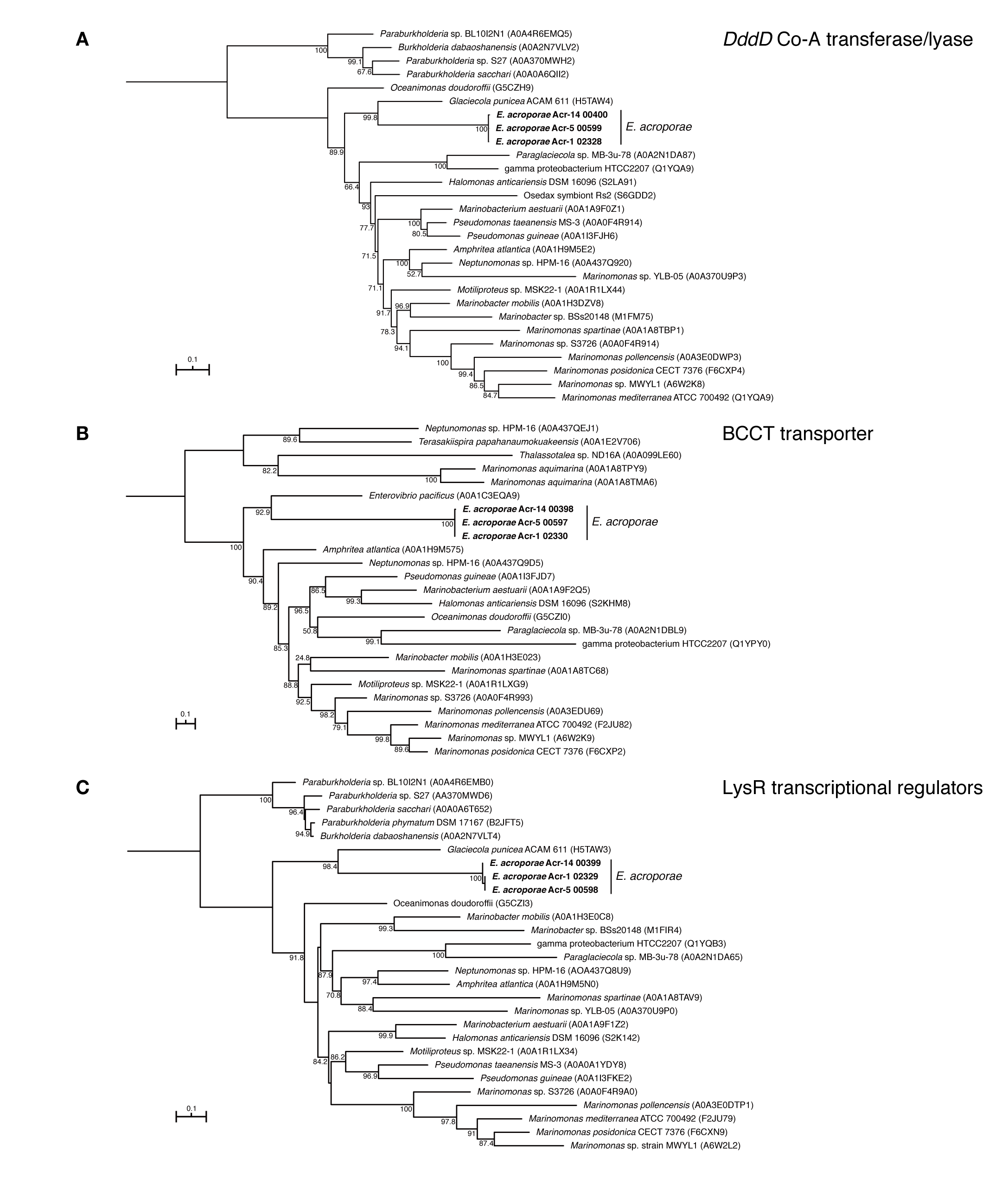

Supplement: Supplementary file 9 — Supplementary Figure S8 [file 41396_2020_610_MOESM9_ESM.png]

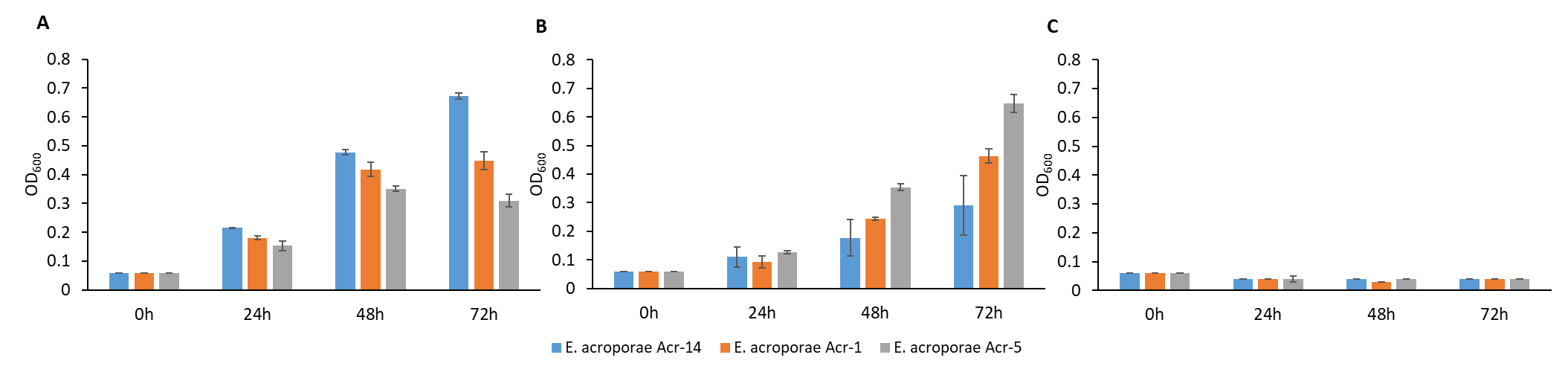

Supplement: Supplementary file 10 — Supplementary Figure S9 [file 41396_2020_610_MOESM10_ESM.tif]

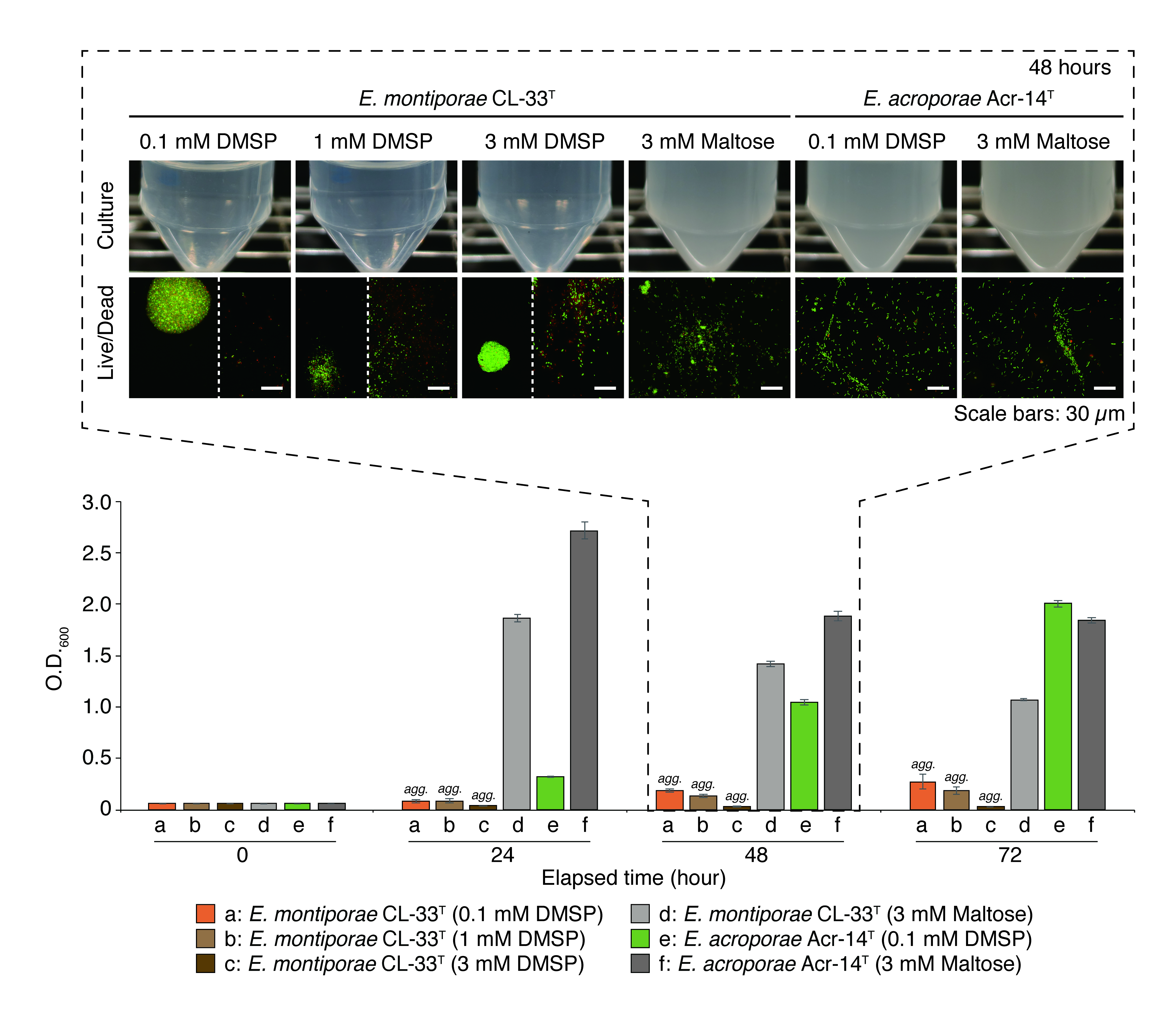

Supplement: Supplementary file 11 — Supplementary Figure S10 [file 41396_2020_610_MOESM11_ESM.tif]
