# Supplementary material for: Comparative genomics: Dominant coral-bacterium Endozoicomonas acroporae metabolizes dimethylsulfoniopropionate (DMSP)
Source: ISME J. 2020 Feb 13;14(5):1290–303. doi: 10.1038/s41396-020-0610-x (PMC7174347; doi:10.1038/s41396-020-0610-x)
Supplement: Supplementary file 12 — Supplementary Table S1 [file 41396_2020_610_MOESM12_ESM.docx]

Supplementary Table S1. Model selection statistics for 16S rRNA gene and core-genome based phylogenetic analysis. The best model was selected based on BIC values.

| **16S rRNA Gene** | | | **Core-genome** | | |
| --- | --- | --- | --- | --- | --- |
| **Model** | **BIC** | **lnL** | **Model** | **BIC** | **lnL** |
| **TIM3+F+I+G4** | 14866.91 | -6940.83 | **GTR+F+I+G4** | 3761748.56 | -1880649.68 |
| TIM2+F+I+G4 | 14867.54 | -6941.14 | SYM+I+G4 | 3462972.64 | -1881280.93 |
| TN+F+I+G4 | 14870.32 | -6946.23 | TIM2+F+I+G4 | 3763150.12 | -1881363.76 |
| GTR+F+I+G4 | 14871.78 | -6935.85 | TIM+F+I+G4 | 3763289.04 | -1881433.22 |
| TIM+F+I+G4 | 14877.29 | -6946.01 | TIMe+I+G4 | 3763577.29 | -1881596.56 |

BIC: Bayesian Information Criterion; lnL: Maximum Log-likelihood; TIM3: Transition model AC=CG, AT=GT, and unequal base frequency; TIM2: Transition model AC=AT, CG=GT, and unequal base frequency; TN: Tamura and Nei model or TN93; GTR: General Time reversible; TIM: Transition models, AC=GT, AT=CG, and unequal base frequency; SYN: Symmetric model with unequal rates but equal base frequency; TIMe: TIM-like, but equal base frequency. F; Empirical codon frequencies counted from the data; I+G4: invariable site plus discrete Gamma model.
