# Supplementary material for: Comparative genomics: Dominant coral-bacterium Endozoicomonas acroporae metabolizes dimethylsulfoniopropionate (DMSP)
Source: ISME J. 2020 Feb 13;14(5):1290–303. doi: 10.1038/s41396-020-0610-x (PMC7174347; doi:10.1038/s41396-020-0610-x)
Supplement: Supplementary file 15 — Supplementary Table S4 [file 41396_2020_610_MOESM15_ESM.docx]

Supplementary Table S4. Genome assembly characteristics of *E. acroporae* strains (this study).

| **Genome Characteristic** | ***E. acroporae*  Acr-14^T^** | ***E. acroporae*  Acr-5** | ***E. acroporae* Acr-1** |
| --- | --- | --- | --- |
| Size (Mbp) | 6.048 | 6.034 | 6.024 |
| GC content | 49.16% | 49.3% | 49.2% |
| N50 | 47,658(bp) | 52,448(bp) | 56,565(bp) |
| No. of rRNA | 5 (16S x 1, 5S x 4) | 7 (16S x 1, 5S x 6) | 6 (16S x 1, 5S x 5) |
| No. of tRNA | 81 | 80 | 77 |
| No. of Genes | 5,104 | 5,190 | 5,144 |
| No. of CDS | 5,018 | 5,101 | 5,059 |
| CRISPRs | 4 | 3 | 2 |
| Gene density (genes/ Mb) | 829 | 898 | 839 |
