# Supplementary material for: Comparative genomics: Dominant coral-bacterium Endozoicomonas acroporae metabolizes dimethylsulfoniopropionate (DMSP)
Source: ISME J. 2020 Feb 13;14(5):1290–303. doi: 10.1038/s41396-020-0610-x (PMC7174347; doi:10.1038/s41396-020-0610-x)
Supplement: Supplementary file 16 — Supplementary Table S5 [file 41396_2020_610_MOESM16_ESM.docx]

Supplementary Table S5. List of genomes used in this study for comparative genomic analysis with genome size, host, and presence of *dddD* gene.

| **Genome** | **Genome Size**  **(# contigs)** | **Host** | ***dddD* gene** |
| --- | --- | --- | --- |
| *Endozoicomonas acroporae* Acr-1 (this study)  (SAUT00000000) | 6.024 Mb (299) | Coral (*Acropora* sp.) | **Present** |
| *Endozoicomonas acroporae* Acr-5 (this study)  (SAUU00000000) | 6.034 Mb (295) | Coral (*Acropora* sp.) | **Present** |
| *Endozoicomonas acroporae* Acr-14^T^ (this study)  (PJPV00000000) | 6.049 Mb (309) | Coral (*Acropora* sp.) | **Present** |
| *Endozoicomonas montiporae* CL-33^T^  (CP013251) | 5.430 Mb (1) | Coral (*Montipora aequituberculata)* | Absent |
| *Endozoicomonas montiporae* LMG24815  (JOKG01000000) | 5.602 Mb (20) | Coral (*Montipora aequituberculata)* | Absent |
| *Endozoicomonas atrinae* WP70 ^T^  (LUKQ02000000) | 6.690 Mb (985) | Comb pen shell (*Atrina pectinate*) | Absent |
| *Endozoicomonas elysicola* DSM22380 ^T^  (JOJP01000001) | 5.606 Mb (2) | Sea Slug (*Elysia ornate)* | Absent |
| *Endozoicomonas* sp. AB1  (MDLD01000000) | 4.049 Mb (272) | *Bugula neritina* AB1 | Absent |
| *Endozoicomonas ascidiicola* AVMART05 ^T^  (LUTV01000000) | 6.135 Mb (36) | Ascidians (Tunicata, Ascidiaceae) | Absent |
| *Endozoicomonas ascidiicola* KASP37  (LUTW01000000) | 6.512 Mb (34) | Ascidians (Tunicata, Ascidiaceae) | Absent |
| *Endozoicomonas numazuensis* DSM25634 ^T^  (JOKH01000000) | 6.340 Mb (31) | Marine sponge | Absent |
| *Endozoicomonas arenosclerae* Ab112 ^T^  (LASA01000000) | 6.453 Mb (328) | Marine sponge (*Arenosclera brasiliensis)* | Absent |
| *Endozoicomonas arenosclerae* E-MC227  (LASB01000000) | 6.216 (2501) | Marine sponge (*Arenosclera brasiliensis)* | Absent |
