# Supplementary material for: Comparative genomics: Dominant coral-bacterium Endozoicomonas acroporae metabolizes dimethylsulfoniopropionate (DMSP)
Source: ISME J. 2020 Feb 13;14(5):1290–303. doi: 10.1038/s41396-020-0610-x (PMC7174347; doi:10.1038/s41396-020-0610-x)
Supplement: Supplementary file 17 — Supplementary Table S6 [file 41396_2020_610_MOESM17_ESM.docx]

Supplementary Table S6. Count of Type III secretion system (T3SS) and Type IV secretion system (T4SS) genes identified in genomes of *Endozoicomonas* species.

| **Genome** | **T3SS gene count** | **T4SS gene count** |
| --- | --- | --- |
| *Endozoicomonas acroporae* Acr-1 (this study) | 499 | 185 |
| *Endozoicomonas acroporae* Acr-5 (this study) | 499 | 186 |
| *Endozoicomonas acroporae* Acr-14^T^ (this study) | 523 | 182 |
| *Endozoicomonas montiporae* CL-33^T^ | 249 | 203 |
| *Endozoicomonas montiporae* LMG24815 | 258 | 187 |
| *Endozoicomonas atrinae* WP70^T^ | 381 | 212 |
| *Endozoicomonas elysicola* DSM22380^T^ | 314 | 182 |
| *Endozoicomonas* sp. AB1 | 165 | 106 |
| *Endozoicomonas ascidiicola* AVMART05^T^ | 343 | 209 |
| *Endozoicomonas ascidiicola* KASP37 | 360 | 226 |
| *Endozoicomonas* *numazuensis* DSM25634^T^ | 301 | 206 |
| *Endozoicomonas arenosclerae* Ab112^T^ | 297 | 203 |
| *Endozoicomonas arenosclerae* E-MC227 | 309 | 181 |
