# Supplementary material for: Comparative genomics: Dominant coral-bacterium Endozoicomonas acroporae metabolizes dimethylsulfoniopropionate (DMSP)
Source: ISME J. 2020 Feb 13;14(5):1290–303. doi: 10.1038/s41396-020-0610-x (PMC7174347; doi:10.1038/s41396-020-0610-x)
Supplement: Supplementary file 18 — Supplementary Table S7 [file 41396_2020_610_MOESM18_ESM.docx]

Supplementary Table S7. Number of Ankrin repeat- and WD40 domain-containing proteins identified in genomes of *Endozoicomonas* *acroporae* strains.

| **Genome** | **Ankyrin repeat proteins** | **WD40 domain proteins** |
| --- | --- | --- |
| *Endozoicomonas acroporae* Acr-1 | 91 | 21 |
| *Endozoicomonas acroporae* Acr-5 | 93 | 21 |
| *Endozoicomonas acroporae* Acr-14^T^ | 92 | 22 |
