# Supplementary material for: Comparative genomics: Dominant coral-bacterium Endozoicomonas acroporae metabolizes dimethylsulfoniopropionate (DMSP)
Source: ISME J. 2020 Feb 13;14(5):1290–303. doi: 10.1038/s41396-020-0610-x (PMC7174347; doi:10.1038/s41396-020-0610-x)
Supplement: Supplementary file 20 — Supplementary Table S9 [file 41396_2020_610_MOESM20_ESM.docx]

| **DddD** | | | **LysR** | | | **BCCT** | | |
| --- | --- | --- | --- | --- | --- | --- | --- | --- |
| **Model** | **BIC** | **lnL** | **Model** | **BIC** | **lnL** | **Model** | **BIC** | **lnL** |
| **LG+I+G4** | 38711.19 | -19168.68 | **LG+I+G4** | 16334.48 | -8007.68 | **LG+G4** | 25506.13 | -12600.11 |
| LG+G4 | 38743.91 | -19188.64 | LG+G4 | 16335.72 | -8011.20 | LG+I+G4 | 25511.41 | -12599.56 |
| WAG+I+G4 | 38836.30 | -19231.27 | JTT+I+G4 | 16375.82 | -8028.34 | LG+F+G4 | 25554.02 | -12563.5 |
| WAG+G4 | 38877.11 | -19255.04 | JTT+G4 | 16376.16 | -8031.42 | LG+F+I+G4 | 25559.13 | -12562.87 |
| LG+F+I+G4 | 38882.14 | -19189.59 | WAG+I+G4 | 16436.33 | -8058.60 | CpREV+F+G4 | 25601.43 | -12587.21 |

Supplementary Table S9. Model selection statistics for DddD, LysR, and BCCT protein sequences for phylogenetic analysis. The best model (in bold) was selected based on BIC values.

BIC: Bayesian Information Criterion; lnL: Maximum Log-likelihood; LG: An improved general amino acid replacement matrix; G: Discrete Gamma distribution; I: Invariable sites; WAG: General empirical model for protein evolution; F: amino-acid frequencies; JTT: Jones-Taylor-Thorton; cpREV: General ReversiblE Chloroplast.
