# Supplementary material for: Comparative genomics: Dominant coral-bacterium Endozoicomonas acroporae metabolizes dimethylsulfoniopropionate (DMSP)
Source: ISME J. 2020 Feb 13;14(5):1290–303. doi: 10.1038/s41396-020-0610-x (PMC7174347; doi:10.1038/s41396-020-0610-x)
Supplement: Supplementary file 4 — Supplementary Figure S3 [file 41396_2020_610_MOESM4_ESM.pdf]

IS element	ISEc46	ISEcret1	ISEc42	ISPa18	IS1618	ISAbe14	ISAeme8	ISAeme9	ISCARN97	ISEcret6	ISEnu2	ISKpn25	ISRop1	ISXne4	ISPa38	ISPsy41	ISShfr7	ISShwo5	ISCARN4	ISEcret9	ISNtsp2	ISPin5
Acr-1	1	2		1	2	1	4	3	1	3	2	1	1	2	1	1	1	1				
Acr-5	1	2		1	2	1	4	3	1	3	1	2	1	1	1	1	1	1				
Acr-14^T	1	3	1	3	1	1	2	9	2	2	2	2	1	5								
CL-33^T	2	1	1																3	4	5	3
LMG-24815	2	1	1	1															4	3	3	2

IS element	ISPlu2	ISPpr13	ISShes1	ISSod15	ISSpu1	ISSpu5	ISThma1	ISPlu19	ISSm4	ISBps1	ISCARN64	ISGes1	ISGur6	ISHbo2	ISHhu3	ISPa82	ISPsy44	ISEcret2	ISEc18	ISEc40	ISPlu3	ISVa15
Acr-1										1	1											
Acr-5																						
Acr-14^T												1	5	1	1	1	1					
CL-33^T	3	7	1	1	1	3	5	5	1									1				
LMG-24815	2	5	1	1	1	3	3	3	1										1	4	1	1

Shared in all

Between *E. acroporae* and *E. montiporae*

Shared in *E. acroporae*

Only shared in *E. acroporae* Acr-1 and Acr-5

Only shared in *E. montiporae*

Unique in a strain
